# Hypothyroidism-induced Rhabdomyolysis in a Pediatric Patient

**DOI:** 10.1210/jcemcr/luae118

**Published:** 2024-07-30

**Authors:** Hend Abd El Baky, Danika Cziranka-Crooks, Brinda Prasanna Kumar, Meghan Jacobs, Jeremy Killion, Lucy D Mastrandrea

**Affiliations:** Department of Pediatrics, Jacobs School of Medicine and Biomedical Sciences, State University of New York at Buffalo, John R. Oishei Children’s Hospital, Buffalo, NY 14203, USA; Department of Pediatrics, Jacobs School of Medicine and Biomedical Sciences, State University of New York at Buffalo, John R. Oishei Children’s Hospital, Buffalo, NY 14203, USA; Department of Pediatrics, Jacobs School of Medicine and Biomedical Sciences, State University of New York at Buffalo, John R. Oishei Children’s Hospital, Buffalo, NY 14203, USA; Department of Pediatrics, Jacobs School of Medicine and Biomedical Sciences, State University of New York at Buffalo, John R. Oishei Children’s Hospital, Buffalo, NY 14203, USA; Department of Pediatrics, Jacobs School of Medicine and Biomedical Sciences, State University of New York at Buffalo, John R. Oishei Children’s Hospital, Buffalo, NY 14203, USA; Department of Pediatrics, Jacobs School of Medicine and Biomedical Sciences, State University of New York at Buffalo, John R. Oishei Children’s Hospital, Buffalo, NY 14203, USA

**Keywords:** hypothyroidism, rhabdomyolysis, myositis, creatine kinase

## Abstract

Hypothyroidism is a common clinical condition with nonspecific symptoms such as fatigue, cold intolerance, and constipation. Rarely, severe primary hypothyroidism presents with rhabdomyolysis. We present a 12-year-old boy with several months of fatigue, muscle cramping, and elevated creatine kinase (CK) who was found to have severe primary hypothyroidism. Initial laboratory evaluation was significant for CK 2056 U/L (reference, 0-300 U/L; 34.34 µkat/L) and creatinine 1.39 mg/dL (reference, 0.4-1 mg/dL; 122.88 µmol/L). He was admitted for management of rhabdomyolysis with acute kidney injury. Further biochemical testing revealed profound hypothyroidism—thyrotropin 494 mIU/mL (reference, 0.40-6.00 mIU/mL) and free thyroxine (T4) less than 0.4 ng/dL (reference, 0.80-1.80 ng/dL; <5.15 pmol/L). Thyroglobulin and thyroid peroxidase autoantibodies were positive, confirming autoimmune hypothyroidism. Low-dose levothyroxine was initiated. With aggressive rehydration, creatinine and CK levels improved. The patient was discharged home with instructions to escalate thyroid hormone replacement over 8 weeks. While the etiology of CK elevation in severe hypothyroidism is poorly understood, it is hypothesized that T4 deficiency alters mitochondrial oxidative capacity and glycogenolysis precipitating muscle atrophy and breakdown with CK release. This case highlights that clinicians should consider thyroid function testing in patients with symptoms of muscle pain and unexplained elevations in CK.

## Introduction

Hypothyroidism is a common clinical condition that results from inadequate production of thyroid hormones or inadequate action of thyroid hormones in target tissues. If left untreated, hypothyroidism can lead to serious adverse effects on multiple organ systems. Acquired primary hypothyroidism is the most prevalent form worldwide with iodine deficiency and Hashimoto thyroiditis as the main causes (1). The clinical picture varies, with presentations ranging from asymptomatic to overt. Those with symptoms may present with fatigue, cold intolerance, and constipation (1). Complaints of muscle stiffness and myalgia may be elicited from patients with undiagnosed hypothyroidism (2). When measured, muscle enzymes may be elevated, but are usually less than 10 times the upper limit of normal (3), and rhabdomyolysis is rare (2). In most of the reported cases of rhabdomyolysis, a precipitating factor has been identified such as strenuous exercise (4) or the use of lipid-lowering drugs (5-8). Rhabdomyolysis associated with hypothyroidism without an obvious precipitating factor has been previously reported in the literature but mostly in adults (3, 9-11). We describe the case of a prepubertal child presenting with rhabdomyolysis caused by profound primary hypothyroidism.

## Case Presentation

A 12-year-old boy with past medical history of asthma, exogenous obesity, and sleep apnea presented to the emergency room after an episode of severe painful muscle cramping preceded by a 3-month history of generalized muscle cramping. Initially, the muscle cramping was noted in the lower extremities, then gradually progressed to involve the lower back and chest. The patient was previously evaluated by his primary care pediatrician, who attributed the symptoms to growing pains and dehydration. He was instructed to increase water intake and start a multivitamin. His history was negative for increased physical exertion, environmental heat exposure, recent illnesses, illicit substances, or supplements. The patient was not on any daily medications. Review of systems was positive for cold intolerance, constipation, pale skin tone, and weight gain. The family did not report dark (tea-colored) urine. Review of his historical growth curve showed that his height had decelerated from the 78th percentile to the 49th percentile over the previous 2 years, while he had gained 45 pounds with a body mass index *Z*-score change from +2.25 to +2.5 over the same period. Family history was significant for autoimmune thyroiditis in his mother and maternal grandmother.

## Diagnostic Assessment

The patient appeared well in the emergency room with no evidence of goiter or edema. His neurologic and musculoskeletal examination was normal including no documented muscle tenderness. His vitals were normal except for a heart rate of 61. Initial laboratory evaluation in the emergency room was significant for a creatinine of 1.39 mg/dL (reference, 0.4-1 mg/dL; 122.88 µmol/L) and creatine kinase (CK) of 2056 U/L (reference, 0-300 U/L; 34.34 µkat/L) (Table 1). Liver enzymes showed mildly elevated aspartate transaminase 62 U/L (reference, 5-50 U/L; 1.04 µkat/L) and normal alanine transaminase 28 U/L (reference, 5-50 U/L; 0.47 µkat/L). Urinalysis was normal and renal ultrasound was obtained, which showed no abnormalities. The patient was admitted for management of acute kidney injury (AKI) secondary to rhabdomyolysis and was started on intravenous fluid hydration. During his first day of admission, he was noted to have bradycardia with a heart rate of 45 bpm. Electrocardiogram showed bradycardia with intraventricular conduction delay. Because of the bradycardia, thyroid function testing were drawn that revealed a thyrotropin (TSH) level of 494 mIU/mL (reference, 0.40-6.00 mIU/mL) with low free thyroxine (T4) of less than 0.4 ng/dL (reference, 0.80-1.80 ng/dL; <5.15 pmol/L). Additional laboratory tests showed strongly positive thyroglobulin 51 U/mL (reference ≤ 4 U/mL; 51 kIU/L) and thyroid peroxidase antibodies 1467 U/mL (reference ≤ 9 U/mL; 1467 kIU/L). Thyroid ultrasound showed a solid and cystic nodule in the inferior left thyroid lobe measuring 1.2 × 1.4 × 1 cm. Laboratory work was also significant for dyslipidemia, mild normocytic anemia, hemoglobin 11.7 gm/dL (reference, 12.5-16.1 g/dL; 117 g/L), and low vitamin D level 14 ng/mL (reference, 30-100 ng/mL; 34.94 nmol/L).

**Table 1.** Laboratory test trend over admission and follow-up

Lab	Day 1	Day 3	7 wk	12 wk	Reference range
T4 free	<0.40 ng/dL (<5.15 pmol/L)				0.80-1.80 ng/dL; (10.30-23.17 pmol/L)
T4			6.80 μg/dL (87.52 nmol/L)	9.60 μg/dL (123.56 nmol/L)	5-11.80 μg/dL (64.36-151.88 nmol/L)
TSH	494.27 mIU/mL		86.06 mIU/mL	8.07 mIU/mL	0.40-6.00 mIU/mL
Creatinine	1.39 mg/dL (122.88 µmol/L)	1.26-1.41 mg/dL (111.38-124.64 µmol/L)	0.81 mg/dL (71.60 µmol/L)	0.67 mg/dL (59.23 µmol/L)	0.40-1 mg/dL (35.36-88.40 µmol/L)
Creatine kinase	2056 U/L (34.34 µkat/L)	1640 U/L (27.39 µkat/L)	164 U/L (2.74 µkat/L)	114 U/L (1.90 µkat/L)	0-300 U/L (0-5.01 µkat/L)
Cholesterol		263 mg/dL (6.81 mmol/L)	163 mg/dL (4.22 mmol/L)		112-208 mg/dL (2.90-5.39 mmol/L)
Cholesterol LDL, calculated		209 mg/dL (5.41 mmol/L)	113 mg/dL (2.93 mmol/L)		0-100 mg/dL (0-2.59 mmol/L)

Values in parentheses are International System of Units (SI).

Abbreviations: LDL, low-density lipoprotein; T4, thyroxine; TSH, thyrotropin.

## Treatment

The patient was managed with aggressive intravenous hydration to treat AKI. He was started on thyroid hormone replacement (levothyroxine) at a dose of 12.5 mcg daily with a plan to escalate to physiologic dose (initial target dose 100 mcg) over the next 8 weeks at a “low and slow” rate to prevent risk of cardiac dysfunction or symptoms of relative hyperthyroidism for the patient. He responded well to the aforementioned treatment and was discharged home on day 4 with recommendations for iron and vitamin D supplementation.

## Outcome and Follow-up

**Figure 1.**
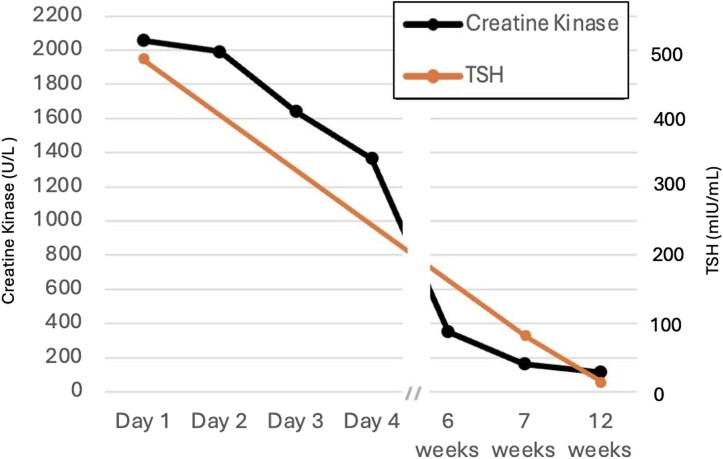
Creatine kinase and thyrotropin (TSH) trend over time.

Outpatient follow-up at 6 weeks showed improvement in CK and creatinine with normalization of levels at 8 weeks (Fig. 1 and Table 1). Repeat thyroid ultrasound conducted 4 months after presentation showed no evidence of the previously identified nodule.

## Discussion

At diagnosis, nearly 80% of patients with severe hypothyroidism will have musculoskeletal symptoms, including easy fatigability and myalgias; these symptoms do not always resolve with initiation of treatment (12). However, rhabdomyolysis is a less well-known muscular manifestation of this disease. CK is the biological marker for muscle inflammation, elevation of CK is common in individuals with hypothyroidism (3, 13), and elevation of CK does not always correlate with myopathic symptoms (14). The etiology of elevated CK secondary to hypothyroidism is not well understood. The mechanism is believed to be due to the fact that T4 deficiency decreases mitochondrial oxidative capacity leading to disruption of glycogenolysis with subsequent atrophy of type 2 muscle fibers that are dependent on glycolysis (2). This atrophy causes muscle breakdown with release of CK and other muscular proteins. In severe cases, this myopathy leads to rhabdomyolysis, which may trigger acute renal failure. Additionally, hypothyroidism leads to decreased renal blood flow with a resulting decline in glomerular filtration rate and consequent increase in creatinine (15).

Rhabdomyolysis is a rare complication of hypothyroidism in adults and even more rare in children. Most patients who develop rhabdomyolysis due to hypothyroidism have an inciting event such as heavy exercise, illness, or medications such as statins (6). Our patient did not have an inciting event but was symptomatic with myalgias over 3 months. This led to symptoms being attributed to viral illness and dehydration, thus delaying the diagnosis of rhabdomyolysis. Additional clinical signs of bradycardia led to further biochemical testing, revealing hypothyroidism as the etiology of rhabdomyolysis. Often with rhabdomyolysis secondary to hypothyroidism, the level of CK is noted to be less than 10 times the normal reference range (16). In this case, the CK was 6.8 times the normal reference range.

Elevated CK levels have been reported in patients receiving treatment for hyperthyroidism despite never having low levels of thyroid hormone (17). It is hypothesized that rapid correction of hyperthyroidism could result in myopathy similar to hypothyroidism (17). Electromyography may be helpful if treatment of hypothyroidism does not lead to correction of CK and there is concern for polymyositis (12).

Primary treatment for hypothyroidism-induced rhabdomyolysis is levothyroxine repletion. Acutely, hydration will be important to treat symptoms, decrease the CK levels, and prevent worsening AKI. In this case, treatment of hypothyroidism addressed both the muscle pathology and renal dysfunction with a downtrend in creatinine and CK over weeks. Future episodes are unlikely with adequate thyroid hormone replacement. This case highlights that clinicians should consider thyroid function testing in patients with unexplained elevations in CK. A thorough history and tailored evaluation will aid in early diagnosis and optimal treatment.

## Learning Points

- Rhabdomyolysis is a lesser known presentation of hypothyroidism and should be considered when managing patients with elevated CK.
- Although inciting events commonly occur prior to hypothyroidism-induced rhabdomyolysis, they are not always present.
- Treatment of hypothyroidism will improve CK and creatinine levels.

## Contributors

All authors made individual contributions to authorship. H.A. was involved in the introduction section. B.K. was involved in the case presentation, diagnostic assessment, treatment, and outcome and follow-up section. D.C.C. was involved in the discussion section. L.M. was involved in the management and treatment of the patient as well as the preparation and revision of the manuscript. M.J. and J.K. were involved in the management and treatment of the patient. All authors reviewed and approved the final draft.

## Data Availability

Original data generated and analyzed during this study are included in this published article.

## Abbreviations

AKI: acute kidney injury
CK: creatine kinase
T4: thyroxine
TSH: thyrotropin
